# Learning curve analysis for competency assessment in fetoscopic laser photocoagulation for twin–twin transfusion syndrome: A model for middle‐resource settings

**DOI:** 10.1002/pmf2.70462

**Published:** 2026-09-10

**Authors:** Vajiheh Marsoosi, Laleh Eslamian, Sotoudeh Mohammadi, Lida Mohamadpour, May Abiad, Ali Javinani, Fatemeh Shojaeian, Aliakbar Rasekhi, Alireza A. Shamshirsaz

**Affiliations:** ^1^ Department of Obstetrics and Gynecology Shariati Hospital Tehran University of Medical Sciences Tehran Iran; ^2^ Department of Obstetrics and Gynecology Baptist Memorial Hospital for Women Memphis Tennessee USA; ^3^ Department of Obstetrics and Gynecology George Washington University School of Medicine and Health Sciences Washington District of Columbia USA; ^4^ Department of Surgery Johns Hopkins University School of Medicine Baltimore Maryland USA; ^5^ Department of Biostatistics Faculty of Medical Sciences Tarbiat Modares University Tehran Iran; ^6^ Fetal Care and Surgery Center Boston Children's Hospital Harvard Medical School Boston Massachusetts USA

**Keywords:** fetoscopic laser photocoagulation training, learning curve, resource‐limited settings, surgical training, twin–twin transfusion syndrome

## Abstract

**Objective:**

To evaluate the learning curve (LC) and skill acquisition for selective fetoscopic laser photocoagulation (sFLP) using cumulative analysis for twin–twin transfusion syndrome (TTTS) treatment in middle‐resource settings.

**Method:**

Two novices with prior expertise in maternal‐fetal medicine and minimally invasive procedures underwent a four‐phase training program for sFLP. The program included literature reviews, observational fellowships, hands‐on simulator training, and surgeries performed under direct supervision at a large tertiary referral center in Iran. Between October 2018 and May 2023, they independently managed and performed sFLP on 230 patients with TTTS. Performance was evaluated using cumulative sum (CUSUM) and learning curve CUSUM (LC‐CUSUM) analyses.

**Results:**

At birth, among the 228 pregnancies included in the birth survival analysis, 81.5% had at least one surviving twin, including 109 patients (47.8%) with survival of both twins, whereas 42 pregnancies (18.5%) resulted in double fetal demise. The learning curve analysis estimated an overall failure probability of 0.18 (95% confidence interval [CI], 0.13–0.23), with a survival probability of 0.82 (95% CI, 0.77–0.87). For outcomes with at least one survivor, the predefined competency threshold was achieved after the 80th patient, after which acceptable performance was maintained throughout the remainder of the study. For double‐twin survival, the probability of failure was 0.52 (95% CI, 0.41–0.54). The predefined competency threshold was achieved after the 110th procedure, after which acceptable performance was similarly maintained throughout the remainder of the study. These findings demonstrate progressive skill acquisition and operational consistency through structured training and continuous quality control.

**Conclusion:**

Structured training enabled acquisition of procedural competency after 80 procedures for outcomes with at least one survivor and 110 procedures for double‐twin survival. LC‐CUSUM and CUSUM analyses provide an objective framework for monitoring implementation of new fetal therapy programs and may support safe expansion of fetoscopic laser photocoagulation in resource‐constrained settings.

## 1 INTRODUCTION

Twin–twin transfusion syndrome (TTTS) affects approximately 10%–15% of monochorionic twin pregnancies, significantly increasing prenatal mortality and neurological morbidity in affected fetuses [1]. This condition arises from imbalanced blood flow between the fetuses through placental anastomoses, leading to severe complications such as oligo/polyhydramnios sequence, cardiac failure, and the loss of one or both twins [2]. The introduction of fetoscopic laser surgery in the 1990s has significantly improved survival outcomes, with reported survival rates for both twins ranging from 30% to 70% [3, 4]. However, the availability of the sophisticated procedure remains highly restricted, predominantly offered in highly specialized fetal medicine clinics across Europe and North America. This results in a substantial accessibility gap, particularly in the Middle East and other underserved regions. Such disparities underscore the urgent need to expand access to fetoscopic laser surgery and disseminate expertise globally, aiming to ultimately improve outcomes for TTTS patients worldwide [5].

To address this need, an initiative was launched at a large tertiary referral center in Iran to establish the region's first structured fetoscopic laser surgery center designed for underserved settings. The study sought to address existing healthcare disparities by building local capacity for fetoscopic laser photocoagulation (FLP) interventions and enabling the treatment of TTTS within the region. A committed team of clinicians participated in a four‐phased structured learning program. This course included an observational fellowship at advanced centers, followed by hands‐on simulation training, mentorship during supervised procedures, and, ultimately, independent practice of this intricate intervention.

FLP for the management of TTTS is a complex, gradual process that requires both technical proficiency and clinical acumen necessary to ensure safe and effective outcomes [6]. While surgeons refine their approach and improve their ability to anticipate potential complications with experience, the intricate nature of this procedure demands special attention [7]. Despite its crucial role, FLP remains inequitably accessible, highlighting the need for broader availability and improved outcomes through a comprehensive understanding of its learning curve across different settings [8].

The primary objective of this study is to evaluate the learning curve associated with FLP for TTTS at our center, guiding MFM specialists considering this procedure for their patients. Through cumulative sum (CUSUM) and learning curve CUSUM (LC‐CUSUM) analyses, we aim to quantify the procedural milestones required to achieve competency, ultimately bridging the gap between limited access and the broader availability of this life‐saving intervention. To validate the effectiveness of our structured training program, we compared the learning curve of the final phase of independent practice with those reported by pioneering centers in developed countries.

## 2 MATERIALS AND METHODS

### 2.1 Study population

This study included monochorionic diamniotic twin pregnancies diagnosed with stage II—IV TTTS between 16 and 28 weeks of gestation, as well as symptomatic stage I deemed eligible FLP. Patients with inadequate follow‐up (*N* = 3) and triplet pregnancies (*N* = 2) were excluded. Diagnosis and staging were determined using the Quintero classification criteria [9]. Comprehensive counseling was provided regarding the procedure's success rates, potential complications, and alternative management options, including expectant management, selective fetal termination, and amnioreduction.

### 2.2 Operator training

The surgical team consisted of two expert surgeons with extensive experience in both maternal fetal medicine and minimally invasive procedures. Both operators completed a specialized structural training program to attain proficiency in FLP. This included:

1. Observer Fellowship: A 3‐week observational fellowship at a leading fetal surgery center in the United States.
2. Simulator Training: Advanced hands‐on training course in Ireland.
3. Supervised Clinical Training: Following completion of the observational fellowship and simulator‐based training, each operator performed approximately 15 fetoscopic laser procedures under direct on‐site supervision (proctorship) by an experienced fetal surgeon.

These supervised procedures were included in the present analysis. Following this supervised phase, all subsequent procedures were performed independently without direct intraoperative proctor supervision. This comprehensive and meticulous training approach was designed to equip the operators with the skills required for independent practice of FLP while also establishing a sustainable framework for fetal therapy expertise in the region.

### 2.3 Surgical technique and follow‐up

FLP was performed under general or spinal anesthesia. A small skin incision was made at the optimal insertion site, and an 11‐Fr introducer was percutaneously inserted into the amniotic cavity using the Seldinger technique. A standardized 11‐Fr introducer was used for all procedures regardless of gestational age. Smaller‐caliber introducers were not used because the 11‐Fr system provided reliable access for the 3.3‐mm fetoscope while accommodating the laser fiber. For procedures performed before 18 weeks of gestation, the introducer was advanced carefully under continuous ultrasound guidance to minimize trauma to the fetal membranes. A 3.3‐mm fetoscope (11506AA Karl Storz) was then introduced through the catheter. For patients with an anterior placenta, a 11530 KB Curved Fetoscope Sheath was utilized. During the procedure, superficial intertwin placental anastomoses were directly identified and coagulated using 0.4‐ or 0.6‐mm fiber and diode laser (Dornier MedTech GMbH). After removing the fetoscope, amniotic fluid was gradually drained through the sheath until a vertical pool of 6–10 cm was reached to minimize the risk of chorioamniotic membrane separation associated with rapid decompression.

Post‐procedure, patients were admitted for 24 h. They received indomethacin (100 mg rectal suppository every 12 h) or nifedipine (10 mg orally, three times daily) for 2 days, along with ceftriaxone (1 g intravenously twice daily for 24 h). Follow‐up ultrasonography was performed 24 h post‐procedure, and pregnant patients without complications were discharged. Subsequent follow‐ups were scheduled every 2 weeks to monitor for iatrogenic twin anemia polycythemia sequence, TTTS reversal or recurrence, and growth disorders.

Deliveries were performed based on obstetric indications, fetal growth parameters, and Doppler findings, with planned delivery no later than 34–35 weeks of gestation at a tertiary referral center. Fetal, maternal, and neonatal outcomes were systematically recorded.

### 2.4 Ethical consideration

The study adhered to ethical guidelines for human subject research and received approval from the institutional ethical review board (IRB approval number: IR.TUMS.MEDIVINE.REC.1403.264). Prior to participation, informed consent was obtained from all parents enrolled in the study.

### 2.5 Statistical analysis

The primary outcome was double‐twin survival, while the secondary outcomes included survival of at least one fetus, preterm prelabor rupture of membranes, gestational age at delivery, birthweight, and neonatal morbidity and mortality.

CUSUM analysis was used to assess surgical performance over time by tracking procedural outcomes such as either success or failure. This method plots deviations from a predefined performance benchmark. Once competency has been achieved, acceptable performance is considered to be maintained as long as the CUSUM score remains below the predefined decision threshold. LC‐CUSUM test was employed to determine whether a predefined competency threshold had been achieved. Initially, the method assumes that performance has not yet reached adequacy. Competency is confirmed once the LC‐CUSUM plot crosses a predefined threshold, after which standard CUSUM analysis is used to ensure continued performance consistency. If the CUSUM plot does not exceed the predefined threshold, acceptable performance is considered to be maintained [7, 10]. For this study, the benchmark for acceptable performance was based on the largest single‐center experience reported by Diehl et al. (1019 patients), using data from the first 200 procedures as a reference. Because the primary objective was to evaluate institutional program implementation rather than individual surgeon performance, procedures performed by both surgeons were analyzed as a single cohort. The acceptable and unacceptable failure probabilities were set at 0.20 and 0.35 for survival of at least one fetus, and at 0.50 and 0.68 for double‐twin survival, respectively.

## 3 RESULTS

**FIGURE 1:**
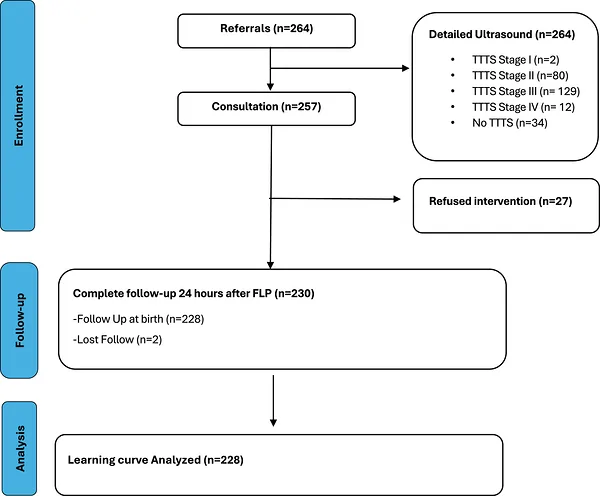
Patient selection flow diagram. FLP, fetoscopic laser photocoagulation; TTTS, twin–twin transfusion syndrome.

A total of 230 patients with survival data 24 h after FLP were included (Figure 1). Gestational age (GA) at intervention ranged from 16 to 28 weeks, with a mean GA at delivery of 31.6 (SD = 4.6) weeks. Table 1 outlines the baseline characteristics of the patients.

**TABLE 1:** Clinical and procedural data at treatment by fetoscopic laser photocoagulation (FLP).

Patient characteristics	Double fetal demise	At least one survivor	Double‐twin survival	Total patients/average
Delivery ≥ 34 weeks, *n* (%)	3 (2.8)	47 (43.9)	57 (53.3)	Total patients: 107 (100)
Maternal age, year, mean (SD)	31.7 (5.9)	29 (5.2)	29.7 (5.1)	29.7 (5.36)
GA at FLP, weeks, mean (SD)	20.6 (2.6)	20.6 (2.0)	21.3 (2.5)	20.9 (2.4)
Cervical length (mm)	30.9 (11.2)	31.4 (8.7)	30.0 (9.6)	30.5 (9.8)
GA at delivery, weeks, mean (SD)	22.76 (8.7)	28.6 (13.2)	31.2 (8)	28.1 (11.4)

Abbreviations: GA, gestational age, SD, standard deviation.

**FIGURE 2:**
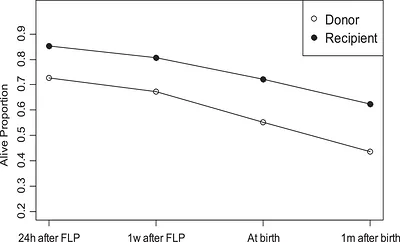
Proportion of surviving individuals at four time points following the fetoscopic laser photocoagulation (FLP) procedure: 24 h post‐FLP, 1 week post‐FLP, at birth, and 1 month post‐FLP.

Survival of recipient and donor twins was evaluated at five different time points, 24 h after FLP, 1 week after FLP, at birth, 30 days after birth, and 6 months after birth (Table 2). Figure 2 illustrates the temporal changes in survival across the follow‐up period. Overall, survival gradually declined over time. At 24 h after the procedure, at least one twin survived in 214 of 230 pregnancies (93.0%), including survival of both twins in 149 pregnancies (64.8%), whereas double fetal demise occurred in 16 pregnancies (7.0%). Birth outcomes were available for 228 pregnancies. At birth, at least one twin survived in 186 pregnancies (81.5%), including survival of both twins in 109 pregnancies (47.8%), whereas double fetal demise occurred in 42 pregnancies (18.5%). Table S1 shows survival at birth based on placental location (anterior or posterior) and TTTS Quintero staging.

**TABLE 2:** Survival outcomes after fetoscopic laser photocoagulation (FLP).

Survival	At least one‐twin survival *n* (%)	Double‐twin survival *n* (%)	Double fetal demise *n* (%)	Total
24 h after FLP	214 (93.0)	149 (64.8)	16 (7.0)	230 (100)
1 week after FLP	204 (90.3)	134 (59.3)	22 (9.7)	226 (100)
At birth	186 (81.5)	109 (47.8)	42 (18.4)	228 (100)
30 days after birth	152 (70.7)	79 (36.7)	63 (29.3)	215 (100)
6 months after birth	150 (70.1)	77 (36.0)	64 (29.9)	214 (100)

### 3.1 Learning curve analysis

#### 3.1.1 At least one survivor

**FIGURE 3:**
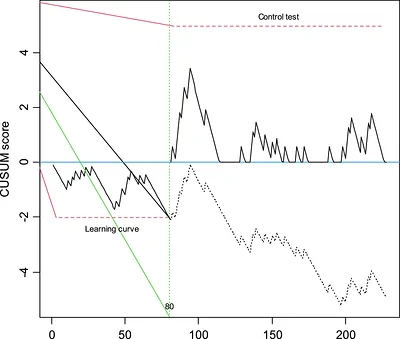
LC‐CUSUM and CUSUM analyses for survival of at least one twin. The predefined competency threshold was reached at the 80th procedure, after which acceptable performance was maintained. The LC‐CUSUM curve is presented on an inverted scale, and its dotted segment represents the hypothetical trajectory had LC‐CUSUM monitoring continued after the competency threshold was reached. CUSUM, cumulative sum; LC‐CUSUM, learning curve cumulative sum.

Learning curves were computed for 228 patients with available survival information at birth. Considering success as the survival of at least one fetus, 186 (81.5%) were deemed successful. This resulted in an estimated failure probability of 0.18, accompanied by a standard error of 0.025. Subsequently, the probability of success was 0.82, with a corresponding 95% confidence interval of (0.77–0.87). Figure 3 shows that acceptable performance was achieved at the 80th procedure and adequacy was maintained afterward.

#### 3.1.2 Double‐twin survivors

**FIGURE 4:**
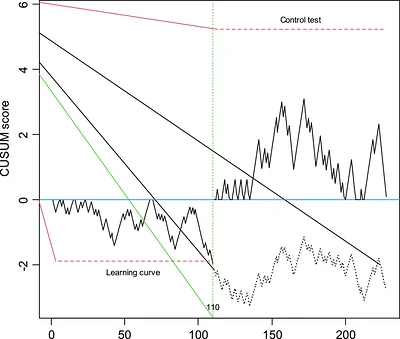
LC‐CUSUM and CUSUM analyses for double‐twin survival. The predefined competency threshold was reached at the 110th procedure, after which acceptable performance was maintained. The LC‐CUSUM curve is presented on an inverted scale, and its dotted segment represents the hypothetical trajectory had LC‐CUSUM monitoring continued after the competency threshold was reached. CUSUM, cumulative sum; LC‐CUSUM, learning curve cumulative sum.

Considering success as double‐twin survival, 109 (47.8%) were successful, with an estimated 𝑝 = 0.52 for failure and a standard error of 0.033. Therefore, the estimated probability of success was 0.48 with a 95% confidence interval (0.41–0.54). Figure 4 shows that acceptable performance was achieved at the 110th procedure and adequacy was maintained afterward.

## 4 DISCUSSION

This study highlights the value of a collaborative approach when introducing a new complex surgical procedure such as selective FLP (sFLP) for TTTS in a middle‐resource setting. Over nearly 5 years, we assessed the acquisition and maintenance of technical performance and demonstrated that these methods provide an objective framework for monitoring surgical performance during program development. Importantly, the predefined competency thresholds achieved after 80 and 110 procedures should not be interpreted as representing the expected clinical performance of a mature high‐volume fetal therapy center. Rather, they represent predefined benchmarks for objectively monitoring technical performance during implementation of a new fetal therapy program. Accordingly, the overall survival observed in this study reflects the entire implementation phase and should not be interpreted as the expected outcomes of an established fetal therapy center.

Our findings align with existing literature. Julian De Lia, the pioneer of TTTS laser surgery, noted a learning curve plateau after 33 procedures [11]. In contrast, Hecher et al. reported a learning curve of 75 procedures, noting a significant improvement in perinatal survival over time [12]. More recently, a prospective observational study from Singapore evaluated the outcomes of sFLP performed by a novice surgical team in three stages: under direct on‐site supervision from an expert mentor, with remote tele‐guidance, and independently at an academic tertiary hospital [13]. The study concluded that systematic mentoring and specialized skills training help new surgeons to navigate the steep learning curve and achieve favorable outcomes when establishing a new practice, particularly in low‐volume settings. Dedicated model training was recommended to develop and maintain the surgical dexterity required for this complex procedure. Pairing this with dedicated model training is crucial for acquiring and sustaining the surgical dexterity needed for this complex procedure.

Consistent with our results, a dual‐center study of 682 participants with diverse TTTS stages reported a 67% survival rate for both twins and an impressive 91% survival rate for at least one twin [14]. Similarly, Morris et al. observed a significant improvement in perinatal survival for at least one twin in a single‐center study of 164 consecutive pregnancies treated with laser coagulation, although the impact of operator experience on double‐twin survival was not assessed [15].

To quantify learning curves, two studies used the CUSUM method. In the first study, among 171 consecutive patients, an observer‐trained operator required 60 procedures to achieve an acceptable success rate, whereas hands‐on trained operators reached this benchmark in just 20 procedures [16]. The latter study, involving 340 consecutive pregnancies, found that four operators attained a satisfactory level of competence after 25–35 procedures [17]. Additionally, a large‐scale study reported that the double‐twin survival rate plateaued at 69% after 600 procedures, this milestone also achieved by a newly trained hands‐on operator who performed 174 of the last 400 procedures [10].

An important implication of our findings is that the learning curve represents a potential burden for patients during the early implementation of a new fetal therapy program. However, its impact may be mitigated through structured stepwise training, simulation‐based practice, mentorship and proctorship during the initial independent cases, standardized surgical protocols, and continuous performance monitoring using LC‐CUSUM and CUSUM analyses. In addition, collaboration between established and emerging fetal therapy centers may facilitate knowledge transfer, accelerate technical skill acquisition, and support the safe dissemination of FLP while minimizing patient exposure to the earliest phase of the learning curve.

Our findings also have important implications for expansion of fetal therapy services in Iran and other middle‐resource settings. Rather than requiring each institution to independently progress through a prolonged learning curve, emerging centers may benefit from partnerships with experienced fetal therapy programs through structured observerships, simulator‐based training, proctored procedures, mentorship, and standardized clinical protocols. At the same time, establishment of new fetal therapy centers should be carefully planned according to regional case volume, institutional infrastructure, and multidisciplinary expertise. Regional referral networks and collaboration between established and emerging centers may therefore provide a more sustainable strategy for expanding access while maintaining procedural quality and patient safety.

The dual fetal demise rate observed in our cohort was higher than that reported by several established high‐volume fetal therapy centers. This finding should be interpreted in the context of implementing the first fetal therapy program for TTTS in Iran. To our knowledge, our institution remains the only center performing FLP for TTTS in Iran and therefore represents the initial national experience rather than a mature high‐volume referral program. Furthermore, differences in case mix, referral patterns, institutional resources, and neonatal intensive care capabilities may also have contributed to variation in outcomes. In particular, differences in neonatal intensive care services and perinatal infrastructure in a middle‐resource setting may influence neonatal survival independently of surgical performance. Therefore, direct comparisons with mature fetal therapy programs should be made with caution.

Another important methodological consideration is the selection of performance benchmarks for CUSUM analysis. In the present study, literature‐based benchmarks derived from a high‐volume expert center were selected because sufficient institutional experience was not yet available to generate reliable site‐specific benchmarks. Once a program has accumulated sufficient experience, however, site‐specific benchmarks may provide a valuable complementary approach for longitudinal performance monitoring by accounting for local case mix and referral patterns. Importantly, such benchmarks are intended to complement, rather than replace, accepted clinical standards established by the literature.

This study has several limitations. First, procedures performed by both surgeons were analyzed as a pooled institutional learning curve; therefore, individual surgeon‐specific learning curves were not evaluated. Because both surgeons completed the same structured training program, followed standardized surgical protocols, and practiced within the same multidisciplinary fetal therapy program, the pooled analysis was considered to best reflect implementation of the institutional program. Nevertheless, this approach may obscure individual variation in learning trajectories. Second, the learning curve was assessed primarily using fetal survival outcomes. Although operative duration and perioperative outcomes may provide complementary information regarding procedural performance, operative time was not evaluated because it is substantially influenced by case‐specific anatomical factors, including placental location, placental vascular anatomy, uterine fibroids, and technical accessibility, independent of surgical proficiency. Furthermore, although survival represents the most clinically relevant patient‐centered outcome following FLP, it is also influenced by multiple factors beyond surgical performance, including disease severity, placental characteristics, gestational age at intervention, and neonatal care. Therefore, survival should be interpreted as an outcome‐based measure of institutional program performance rather than a direct measure of technical proficiency. Finally, this study reflects the experience of only two surgeons from a single center who completed the same structured training program. Therefore, the number of procedures required to achieve competency should not be interpreted as a universal benchmark, as learning curves may vary according to surgeon experience, case complexity, institutional resources, and training pathways. Future multicenter studies incorporating surgeon‐specific analyses and additional standardized technical performance metrics are warranted to further validate the reproducibility and generalizability of these findings.

## 5 CONCLUSION

This study evaluated implementation of a new FLP program for TTTS in a middle‐resource setting using LC‐CUSUM and CUSUM analyses. Competency thresholds were achieved after 80 procedures for survival of at least one twin and 110 procedures for double‐twin survival. These findings support the use of objective performance monitoring during implementation of new fetal therapy programs and highlight the importance of structured training, mentorship, regional collaboration, and continuous quality monitoring to facilitate safe program development.

## CONFLICT OF INTEREST STATEMENT

The authors declare no conflicts of interest.

## FUNDING INFORMATION

The authors received no specific funding for this work.

## Supporting information



Supporting information
