# Supplementary material for: Learning curve analysis for competency assessment in fetoscopic laser photocoagulation for twin–twin transfusion syndrome: A model for middle‐resource settings
Source: Pregnancy (Hoboken). 2026 Sep 10;2(5):e70462. doi: 10.1002/pmf2.70462 (PMC13559468; doi:10.1002/pmf2.70462)
Supplement: Supplementary file 1 [file PMF2-2-e70462-s001.docx]

**Supplementary Table 1**. Survival at birth based on placental location and TTTS Quintero staging**.**

| Placenta Location | | | | Outcome | | | Total |
| --- | --- | --- | --- | --- | --- | --- | --- |
|  |  |  |  | **Double Fetal Demise** | **At Least One Survivor** | **Double-Twin Survival** |  |
| Anterior | TTTS Quintero staging | II | n (%) | 5 (27.8%) | 7 (21.2%) | 16 (36.4%) | 28 |
|  |  | III | n (%) | 16 (72.2%) | 23 (69.7%) | 26 (59.1%) | 65 |
|  |  | IV | n (%) | 0 (0.0%) | 3 (9.1%) | 2 (4.5%) | 5 |
| Posterior | TTTS Quintero staging | I | n (%) | 0 (0.0%) | 0 (0.0%) | 2 (3.1%) | 2 |
|  |  | II | n (%) | 7 (33.3%) | 21 (46.5%) | 26 (39.1%) | 54 |
|  |  | III | n (%) | 12 (57.1%) | 22 (51.2%) | 33 (51.6%) | 67 |
|  |  | IV | n (%) | 2 (9.5%) | 1 (2.3%) | 4 (6.3%) | 7 |
| Total | TTTS Quintero staging | I | n (%) | 0 (0.0%) | 0 (0.0%) | 2 (1.9%) | 2 |
|  |  | II | n (%) | 12 (30.8%) | 28 (35.5%) | 42 (38%) | 82 |
|  |  | III | n (%) | 28 (64.1%) | 45 (59.2%) | 59 (54.6%) | 132 |
|  |  | IV | n (%) | 2 (5.1%) | 4 (5.3%) | 6 (5.6%) | 12 |
|  | Total | | n | 39 | 76 | 108 | 228 |
